# Joint Lossless Image Compression and Encryption Scheme Based on CALIC and Hyperchaotic System

**DOI:** 10.3390/e23081096

**Published:** 2021-08-23

**Authors:** Miao Zhang, Xiaojun Tong, Zhu Wang, Penghui Chen

**Affiliations:** 1 School of Computer Science and Technology, Harbin Institute of Technology, Weihai 264209, China; zm@hitwh.edu.cn (M.Z.); chenpenghui@abchina.com (P.C.); 2 School of Information Science and Engineering, Harbin Institute of Technology, Weihai 264209, China; wangzhu@hit.edu.cn; 3 Agricultural Bank of China, Beijing 100089, China

**Keywords:** lossless image compression and encryption, CALIC, hyperchaotic system, pseudo-random sequence

## Abstract

For efficiency and security of image transmission and storage, the joint image compression and encryption method that performs compression and encryption in a single step is a promising solution due to better security. Moreover, on some important occasions, it is necessary to save images in high quality by lossless compression. Thus, a joint lossless image compression and encryption scheme based on a context-based adaptive lossless image codec (CALIC) and hyperchaotic system is proposed to achieve lossless image encryption and compression simultaneously. Making use of the characteristics of CALIC, four encryption locations are designed to realize joint image compression and encryption: encryption for the predicted values of pixels based on gradient-adjusted prediction (GAP), encryption for the final prediction error, encryption for two lines of pixel values needed by prediction mode and encryption for the entropy coding file. Moreover, a new four-dimensional hyperchaotic system and plaintext-related encryption based on table lookup are all used to enhance the security. The security tests show information entropy, correlation and key sensitivity of the proposed methods reach 7.997, 0.01 and 0.4998, respectively. This indicates that the proposed methods have good security. Meanwhile, compared to original CALIC without security, the proposed methods increase the security and reduce the compression ratio by only 6.3%. The test results indicate that the proposed methods have high security and good lossless compression performance.

## 1. Introduction

With the development of network technology, more and more image files are accessed and shared over the network. To ensure the security of image files, the security protection on images is very important. An efficient way to protect images is image encryption. In recent years, due to the good cryptography properties of chaos, chaos-based image encryption methods have been proposed [1,2,3,4,5,6,7,8,9,10,11]. Masood et al. [10] used three-dimensional Lorenz chaotic maps for diffusion processes. Zhang et al. [11] proposed several image encryption schemes for popular image formats based on chaotic maps. However, the chaotic map is a low-dimensional chaotic system, and the key space is limited. Due to a larger key space and more complex dynamical characteristics, the hyperchaotic system is introduced to image encryption [12,13,14,15,16]. Tong et al. [14,15] proposed a new four-dimensional hyperchaotic system to achieve image encryption. However, the maximum Lyapunov exponent of the new four-dimensional hyperchaotic system is small. In Ref. [14], selected chaotic sequences based on plain image pixels at the fixed position are vulnerable to attack. Moreover, due to the failed design of the encryption process, some of these methods are insecure [17,18].

In the environment of information explosion, the image also occupies a large amount of storage space. To improve the efficiency of image transmission and storage, the effective compression of the image is very important. Therefore, it is necessary to research image compression and encryption. The traditional method is to divide the process into two steps: encryption and compression. Encryption-then-Compression (ETC) and Compression-then-Encryption (CTE) are two ways [19]. For example, in Ref. [20], the red (R), green (G) and blue (B) sub-bands of images are compressed using lossless techniques separately. Then the compressed results are encrypted by employing the idea of chaotic shift encoding (CSK) modulation. However, if compression and encryption are carried out separately, an attacker can attack encryption directly while ignoring compression. Therefore, a joint compression and encryption scheme that performs compression and encryption in a single step is a promising method.

Many joint image compression and encryption algorithms have been proposed [21,22,23,24,25,26,27,28,29,30,31,32,33,34,35,36,37,38,39,40,41,42,43,44]. Among them, key-controlled operations in entropy coding methods have become a research direction [21,22,23,24,25]. In these methods, encryption is embedded into entropy coding, such as arithmetic coding and Huffman coding. The basic idea is to use the key to control the statistical model of entropy coding. Such an entropy encoder can be easily converted into a cryptosystem. Usama et al. [21] proposed secure adaptive Huffman coding which introduces a key control in the compression and decompression processes of the adaptive Huffman coding. Zhao et al. [24] used the encryption key to change the symbol orders in the coding interval and then change the coding interval and code word of traditional arithmetic coding. In Ref. [26], encryption is not embedded into entropy coding, but scrambling encryption is performed before arithmetic coding. However, due to not considering the characteristics of the image, entropy coding method is directly applied to image compression and the compression ratio is relatively low.

In recent years, with the development of compressive sensing (CS), joint image compression and encryption methods based on CS have been proposed [27,28,29,30,31,32]. Zhou et al. [27] proposed the first joint image compression and encryption algorithm based on CS. However, methods based on CS realize lossy compression. On some important occasions such as in aerospace, remote sensing and medicine, higher quality images are required. These occasions not only need high image security, but also need to restore the image completely after compression. Therefore, it is very necessary to research the joint lossless image compression and encryption method.

In addition, there are some joint methods of image encryption and compression based on sparse decomposition [15,33,34]. For example, Tong et al. [15] employed the discrete cosine transformation dictionary to sparsely represent the color image to achieve image compression and encryption simultaneously. However, it performs lossy compression. These methods all perform lossy compression.

With the development of quantum computing, some quantum image compression and encryption algorithms have been proposed [35,36]. Zhou et al. [35] proposed quantum image compression and encryption algorithms with Daubechies *D*^(4)^ quantum wavelet transform (DQWT). Li et al. [36] proposed a quantum image compression-encryption scheme based on quantum discrete cosine transform. However, they perform lossy compression.

The joint image compression and encryption in the transformation coding is also a research hotspot [37,38,39,40,41,42,43,44]. Ye et al. [38] proposed a multi-image compression-encryption scheme based on quaternion discrete fractional Hartley transform. Zhang et al. [43] employed curvelet transform to achieve joint image compression and encryption. However, they perform lossy compression. Moreover, the method proposed by Zhang et al. [43] has low coding efficiency. There are also very few methods to perform lossless compression, such as the method proposed by Zhang et al. [39] and Tong et al. [44]. Zhang et al. [39] proposed lossless image compression and encryption based on set partitioning in hierarchical trees (SPIHT) and cellular automata. Tong et al. [44] employed integer wavelet transform and encrypted multiple rounds in the process of wavelet coefficients and SPIHT.

The context-based adaptive lossless image codec (CALIC) is a kind of adaptive lossless image compression standard [45] which proposes a prediction model based on gradient adjustment. The codec obtains higher lossless compression of continuous-tone images than the UCM (Universal Context Modeling) method. The implementation of the CALIC algorithm is simple, involving mostly integer arithmetic and simple logic. In the application scenarios with high image correlation, compared with lossless compression and encryption methods based on frequency domain transformation proposed in Refs. [39,44], the compression ratio can be greatly improved by using prediction mode. In addition, the compression method based on prediction mode only transmits and encodes the final prediction error and the predicted values of pixels. The coding does not involve more complex operations and is suitable for hardware with less complex computation. In this paper, a joint lossless image compression and encryption scheme based on CALIC and hyperchaotic system is designed, in which the encryption algorithm is embedded in four locations of the compression process. A new hyperchaotic system with larger maximum Lyapunov exponent is constructed to perform encryption. The encryption process with a hyperchaotic system is designed according to the characteristics of CALIC to ensure compression efficiency. Moreover, plaintext-related encryption based on table lookup is used to enhance the security. This paper is organized as follows: Section 2 describes the principles of CALIC lossless image compression and gives an analysis for the encryption locations in CALIC. Section 3 illustrates the joint compression and encryption scheme in detail. Section 4 analyses the compression efficiency and security of proposed algorithm. Finally, Section 5 provides a conclusion for this paper.

## 2. Research on Encryption Locations for CALIC

Firstly, we research the principles of CALIC. Then based on the principle of CALIC, feasibility analyses of the encryption locations are given.

### 2.1. Brief Description of CALIC

CALIC is a sequential coding scheme which encodes and decodes in raster scan order. It uses prediction and context templates only involving the two previous scan lines of coded pixels. Two-stage adaptive prediction scheme via context modeling of errors and error feedback are used in CALIC. 

CALIC is operated in two modes: binary and continuous tone. Binary mode is for situations in which the current locality of the input image has no more than two distinct intensity values; hence it is designed for a more general class of images than the traditional class of black and white images. The system selects one of the two modes during the coding process, depending on the context of the current pixel. Mode selection is automatic and completely transparent to the user. The detailed descriptions of the two modes are as follows.

#### 2.1.1. Continuous-Tone Mode

In continuous-tone mode, the system has four major integrated components: gradient-adjusted prediction (GAP), context selection and quantization, context modeling of prediction errors, and entropy coding of prediction errors. Generation of prediction errors and coding process are as follows.

1. Gradient-adjusted prediction (GAP)

**Figure 1:**
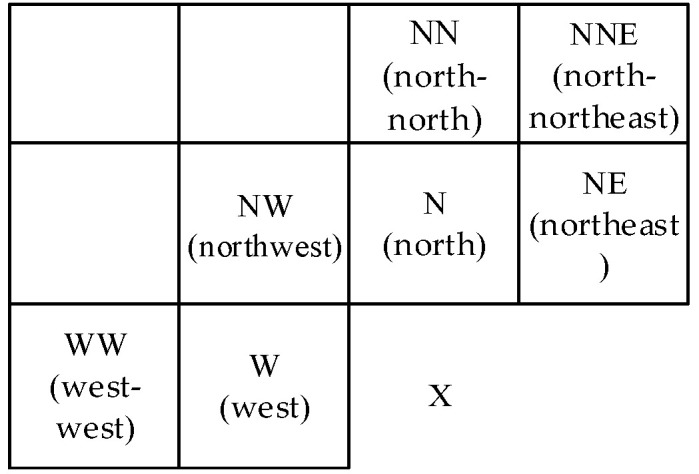
Labeling of neighboring pixels used in GAP.

GAP predictor can adapt itself to the intensity gradients near the predicted pixel. One predicted value requires seven pixels around it. They are on the north, west, northeast, northwest, north-north, west-west, and north-northeast of the pixel. Figure 1 shows the location of the seven pixels.

The neighboring pixels are shown as [45]:

(1) $$\begin{array}{l} I_{N}=I[i,j-1],I_{W}=I[i-1,j],I_{NE}=I[i+1,j-1],\\ I_{NW}=I[i-1,j-1],I_{NN}=I[i,j-2],I_{WW}=I[i-1,j],\\ I_{NNE}=I[i+1,j-2]. \end{array}$$

The gradient of the intensity function at the current pixel by the following quantities [45]:

(2) $$\{\begin{array}{l} d_{h}=\left|I_{W}-I_{WW}\right|+\left|I_{N}-I_{NW}\right|+\left|I_{NE}-I_{N}\right|\\ d_{v}=\left|I_{W}-I_{NW}\right|+\left|I_{N}-I_{NN}\right|+\left|I_{NE}-I_{NNE}\right| \end{array}.$$

In Equation (2), *d_h_* and *d_v_* are estimates of the gradients of the intensity function near the pixel *I*[*i*,*j*] in the horizontal and vertical directions. According to Equation (2), the current pixel value can be predicted, and the prediction algorithm is shown in Algorithm 1 [45].
**Algorithm 1** Pixel gradient prediction algorithm**Input** Original pixels *I* **Output**  The predicted value *Î* (a gradient-adjusted prediction *Î* of *I*) 1. if (*d_v_* − *d_h_* > 80){sharp horizontal edge} $\hat{I}[i,j]=I_{W}$ 2. else (*d_v_* − *d_h_* < −80){sharp vertical edge} $\hat{I}[i,j]=I_{N}$ 3. else { 4. $\hat{I}[i,j]=(I_{W}+I_{N})/2+(I_{NE}-I_{NW})/4$ 5. if (*d_v_* − *d_h_* > 32){ horizontal edge} $\hat{I}[i,j]=(\hat{I}[i,j]+I_{W})/2$ 6. else if (*d_v_* − *d_h_* > 8){weak horizontal edge} $\hat{I}[i,j]=(3\hat{I}[i,j]+I_{W})/4$ 7. else if (*d_v_* − *d_h_* < −32){vertical edge} $\hat{I}[i,j]=(\hat{I}[i,j]+I_{N})/2$ 8. else if (*d_v_* − *d_h_* < −8){weak vertical edge} $\hat{I}[i,j]=(3\hat{I}[i,j]+I_{N})/4$ 9. }

2. The predicted values of pixels are used for coding context selection and quantization. Coding context selection and quantization

The prediction step does not completely remove the statistical redundancy in the image. The variance of prediction errors $e=I-\hat{I}$ strongly correlates to the smoothness of the image around the predicted pixel *I*[*i*,*j*]. To model this correlation at a small computational cost, an error energy estimator is defined as follows [45]:

(3) $$\Delta =ad_{h}+bd_{v}+c\left|e_{w}\right|.$$

where *e_w_* is the previous prediction error, $e_{w}=I[i-1,j]-\hat{I}[i-1,j]\;,\;\;\;\;a=b=1,c=2$. Quantize Δ to *L* levels. *L* = 8 is found to be sufficient in practice. Denote the Δ quantizer by Q, i.e., Q:Δ→{0,1,2,...,7}. The quantization criterion is to minimize conditional entropy of errors. According to the definition of entropy, such that [45]

(4) $$-\underset{e}{\sum }p(e)\log p(e|q_{d}\leq \Delta \leq q_{d}+1).$$

is minimized, where $0=q_{0}<q_{1}<...<q_{L-1}<q_{L}=\infty$ is used to part Δ into L intervals. When *a* = *b* = 1, *c* = 2, after training using all ISO test images, the value of the variable *q* can be determined as follows [45].

(5) $$\begin{array}{l} q_{1}=5,q_{2}=15,q_{3}=25,q_{4}=42,\\ q_{5}=60,q_{6}=85,q_{7}=140 \end{array}$$

Thus, error energy estimator is put into error energy quantizer, which makes preparation for context modeling of prediction errors.

3. Context modeling and prediction errors

Some of the relationships between the predicted pixel *I*[*i*,*j*] and its surroundings cannot be characterized by gradients alone. In order to improve the compression performance, context modeling of the predicted error $e=I-\hat{I}$ is performed. Context modeling of the prediction error mainly includes formation and quantification of context, estimation of error magnitude within a context, and error feedback and sign flipping.

- Formation and quantization of contexts

Contexts for error modeling are constructed to form compound contexts by combining 144 texture contexts with four error energy contexts. Texture context *C* includes the neighborhood of pixel values, which is shown as follows [45]:

(6) $$C=\{x_{0},x_{1},x_{2},...,x_{7}\}=\{I_{N},I_{W},I_{NW},I_{NN},I_{WW},2I_{N}-I_{NN},2I_{W}-I_{WW}\}.$$

Then *C* is quantized into an 8-bit binary number *B*, and the quantization rule is shown as follows [45]:

(7) $$b_{k}=\{\begin{array}{c} 0,if\;\;x_{k}\geq \hat{I}[i,j]\\ 1,if\;\;x_{k}<\hat{I}[i,j] \end{array}0\leq k<K=8.$$

After that, quantized texture pattern $B=b_{7}b_{6}...b_{0}$ is formed. 

By combining the quantized error energy with the quantized texture pattern, compound modeling contexts are determined, denoted by C(δ,β).

- Estimation of error magnitude within a context

The sample mean $\bar{e}(\delta ,\beta )$ in Equation (8) for different compound contexts is used to estimate conditional expectations E{e|C(δ,β)} within each compound context [45].

(8) $$\bar{e}(\delta ,\beta )=S(\delta ,\beta )/N(\delta ,\beta ).$$

where S(δ,β) is the accumulator of e(δ,β). N(δ,β) is the number of occurrences of C(δ,β) for each compound context C(δ,β).

- Error feedback and sign flipping

In a given compound context C(δ,β), correct the bias in prediction by feedback $\bar{e}(\delta ,\beta )$ [45]:

(9) $$\tilde{I}=\hat{I}+\bar{e}(\delta ,\beta ).$$

The final prediction error e is obtained in Equation (10) [45].

(10) $$e=I-\tilde{I}.$$

By checking the symbol of $\bar{e}(\delta ,\beta )$ before encoding e, the symbol of *e* is flipped. Sign flipping is used to improve the code efficiency.

4. Entropy Coding

Entropy coding is performed on final prediction error e and arithmetic coding is used in CALIC.

#### 2.1.2. Binary Mode

When neighboring pixels of *I*[*i*,*j*] have no more than two different values, the predictive coding will have poor performance. The binary mode is used to solve this problem. Let *s*_1_ = *I_W_*, and let other values, if any, be *s*_2_. A ternary code *T* used to describe three states of *I*[*i*,*j*] is defined in Equation (11) [45].

(11) $$T=\{\begin{array}{l} 0\;\;\;\;\;\;\;\;\;\;if\;\;I[i,j]\;\;=\;\;s_{1}\\ 1\;\;\;\;\;\;\;\;\;if\;\;I[i,j]\;\;=\;\;s_{2}\\ 2\;\;\;\;\;\;\;\;\;\;\;\;\;\;otherwise \end{array}.$$

The algorithm switches binary mode to continuous-tone mode in the escape case *T* = 2.

In the binary mode, context prediction model is also needed for the design. It is simpler than continuous-tone mode. Only pixels in six directions are recorded as follows [45].

(12) $$C=\{x_{0},x_{1},...,x_{5}\}=\{I_{W},I_{N},I_{NW},I_{NE},I_{WW},I_{NN}\}.$$

Then *C* is quantized to a 6-bit binary number $B=b_{5}b_{4}\cdots b_{0}$ [45]

(13) $$b_{k}=\{\begin{array}{c} 0\;\;\;\;\;\;\;if\;\;x_{k}=s_{1}\\ 1\;\;\;\;\;if\;\;x_{k}=s_{2} \end{array}0\leq k<6.$$

After quantization, entropy encoding is used to code three symbols.

### 2.2. Feasibility Analysis on Image Encryption Based on CALIC

Through the analysis of the CALIC, we find the possible encryption locations and carry out a feasibility analysis.

#### 2.2.1. Feasibility Analysis of Plaintext Image in Encoder

Plaintext image is the input of the Encoder. The CALIC encoder compresses images in a raster scan order, so the encrypted image can also be coded, and the encryption is feasible. However, the encrypted image destroys the correlation between pixels, which will greatly affect the compression efficiency, so the images can be encrypted but are not desirable.

#### 2.2.2. Feasibility Analysis of the Predicted Values of Pixels

In gradient-adjusted prediction, the predicted value $\hat{I}$ is obtained, and then the error energy is quantized. Encrypting the predicted value $\hat{I}$ can not only impact the subsequent model establishment but also scramble the coding process. Thus, encrypting the predicted value $\hat{I}$ can enhance the security of the compression.

#### 2.2.3. Feasibility Analysis of the Final Predicted Errors

In context modeling and prediction errors, correcting the bias in prediction by feedback forms the final prediction error *e*. The final prediction error is the last output before entropy coding. If encrypting the final prediction error *e*, it will affect the entropy coding which will definitely improve the security of the compressed image files.

#### 2.2.4. Feasibility Analysis of Two Lines of Pixel Values

In CALIC, the first two lines of pixel values of the image as the initial value of coding are finally written into the coding file. Moreover, the first two lines of pixel values after encoding do not perform any operation. Therefore, the information needs to be encrypted to prevent the plaintext information leakage which is feasible.

#### 2.2.5. Feasibility Analysis of Compressed File after Entropy Coding

After the completion of the entropy coding, compressed files can also be encrypted. This is equivalent to encrypting the compressed file, so it is obviously feasible to encrypt this file. Moreover, designing complex encryption algorithms will not affect the compression ratio, which greatly improves the security of the compression algorithm.

Table 1 shows the feasibility of encryption locations and their impact on compression efficiency.

According to the analysis in Section 2, four encryption positions are found, and the corresponding encryption algorithms are designed according to the characteristics of different encryption locations.

## 3. Design of Lossless Image Compression and Encryption Scheme

**Figure 2:**
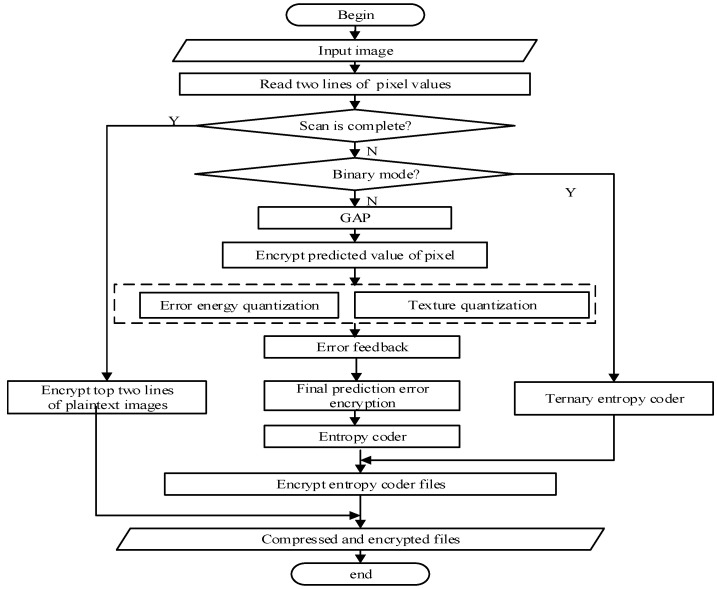
Lossless image compression and encryption scheme.

According to the analysis of the encryption locations of CALIC, we give the flow chart of the image compression and encryption scheme as shown in Figure 2.

According to Figure 2, the four encryption locations are distributed in the CALIC lossless image compression algorithm. To ensure the security, in two modes of CALIC encryption is carried out, respectively. The detailed description of the image compression and encryption scheme is given below.

### 3.1. Design of Pseudo-Random Sequence Generator

#### 3.1.1. Design of Hyperchaotic System

Nonlinear characteristics of chaotic systems can be used in many fields and have made great progress. Chaotic systems have natural links with cryptography systems in initial value sensitivity and long-term unpredictability. In this paper, a new hyperchaotic system is designed based on the Lorenz chaotic system. Compared with a low-dimensional chaotic system, the hyperchaotic system has more complex dynamical characteristics, a larger key space and better randomness. The hyperchaotic system can be used for encryption. Construction rules of the hyperchaotic system are as follows.

1. The necessary condition for the hyperchaotic system based on the Lorenz chaotic system is in differential equations:

(14) $$\frac{dx_{i}}{dt}=f_{i}(x_{1},x_{2},\cdots x_{i},\cdots ,x_{n})\;\;\;\;\;\;\;\;\;\;\;\;i=1,2,...n,n\in N,n>3.$$

where $fi(x_{1},x_{2},...,x_{i},...,x_{n})$ does not contain the term $x_{i}^{k}(k>1)$. In other words, if $fi(x_{1},x_{2},...,x_{i},...,x_{n})$ contains $x_{i}^{k}(k>1)$, this system is not chaotic.

2. Add the nonlinear term. Add a new variable and its first-order dynamic differential equation and introduce its nonlinear term and increase the dimension of the system.
3. Lyapunov function *V* of differential equations satisfies:

(15) $$\nabla V=\frac{\partial {\dot{x}}_{1}}{\partial x_{1}}+\frac{\partial {\dot{x}}_{2}}{\partial x_{2}}+\cdots +\frac{\partial {\dot{x}}_{n}}{\partial x_{n}}<0.$$

Only in this way can the dynamic system produce the dissipative structure and chaotic attractor.

According to the above principle, a new four-dimensional hyperchaotic system is constructed. The new hyperchaotic system introduces a nonlinear term w, which is fed back to the other three equations. The new hyperchaotic system is shown as follows.

(16) $$\{\begin{array}{l} \frac{dx}{dt}=22.35(y-x)+0.01z+w\\ \frac{dy}{dt}=25x+10.7y-xz+3w\\ \frac{dz}{dt}=2x^{2}+0.3y-3.5z+0.1w\\ \frac{dw}{dt}=-1.2x+0.29w\;\;\;\;\;\;\;\;\;\;\;\;\;\;\;\;\;\;\;\;\;\;\;\;\;\;\;\;\;\;\;\;\;\;\;\;\;\;\; \end{array}.$$

#### 3.1.2. Performance Analysis of Hyperchaotic System

1. Lyapunov exponent of Hyperchaotic system

Lyapunov exponent indicates the degree of chaos of the chaos system. The greater the Lyapunov exponent is, the better the chaotic characteristics are. Four Lyapunov exponents of four-dimensional hyperchaotic system satisfy that two Lyapunov exponents are greater than 0, one Lyapunov exponent is 0, and one Lyapunov exponent is less than 0. The Lyapunov exponent test is shown in Table 2.

**Figure 3:**
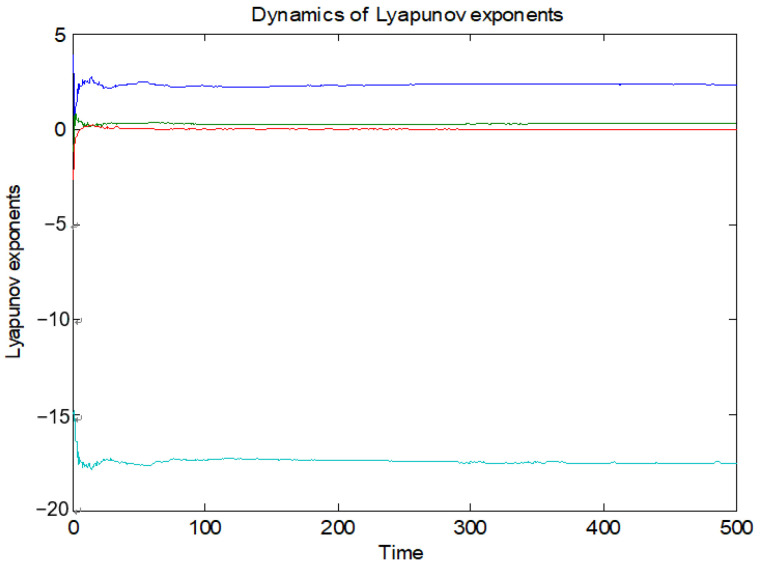
Dynamics of Lyapunov exponents.

In four-dimensional hyperchaotic system, the maximum Lyapunov exponent characterizes the maximum exponential separation rate of the system orbit. Other Lyapunov exponents are ignored because of largest Lyapunov exponent. Therefore, the maximum Lyapunov exponent characterizes the typical characteristics of chaotic system, and it can also show the character of the chaotic system. From Table 2, compared with the Lorenz system, Rossler system and hyperchaotic system mentioned in Refs. [12,13,15,46], we can see that the maximum Lyapunov exponent of the proposed system is larger than that of other systems. Therefore, the complexity of the proposed hyperchaotic system is greater than that of Lorenz system and some conventional hyperchaotic systems. The new hyperchaotic system has a greater Lyapunov exponent. Dynamics of Lyapunov exponents for new hyperchaotic systems are shown in Figure 3.

2. Dissipativity of hyperchaotic system

Dissipativity of hyperchaotic system is $\nabla V=\frac{\partial \dot{x}}{\partial x}+\frac{\partial \dot{y}}{\partial y}+\frac{\partial \dot{z}}{\partial z}+\frac{\partial \dot{w}}{\partial w}$= −22.35 + 10.7 − 3.5 + 0.29 = −14.86 < 0, which indicates that the system is a dissipative system. The volume element of the system shrinks at an exponential rate $\frac{dV}{dt}=e^{-14.86}$. With t→∞, the trajectory of the equation will evolve into an invariant set of attractors.

**Figure 4:**
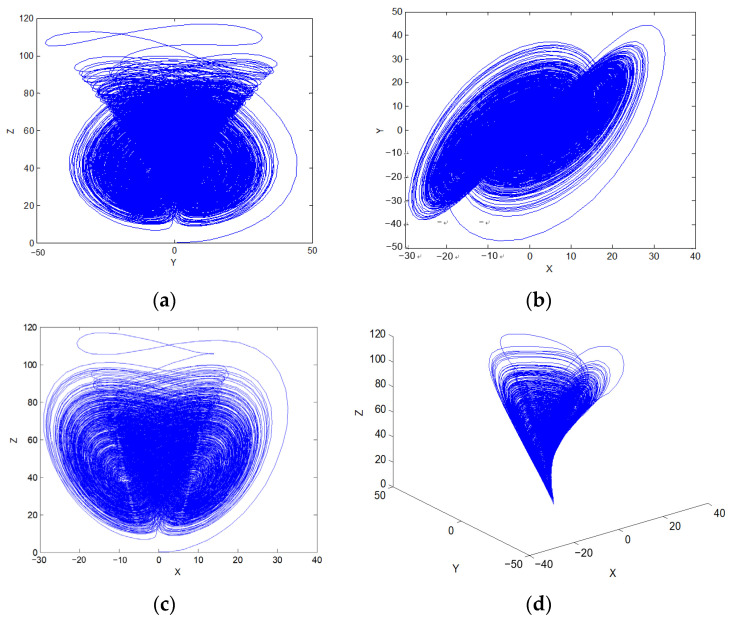
Chaotic attractors and projection on different planes. (**a**) Projection on Y-Z plane; (**b**) projection on X-Y plane; (**c**) projection on X-Z plane; (**d**) projection on X-Y-Z plane.

The hyperchaotic system has two characteristics: contraction and divergence. Contraction is the result of dissipation, and divergence is the result of instability. For a hyperchaotic system, due to the existence of dissipation term, the system will shrink to a limited range. However, in the local orbit, the close orbits will be separated because of mutual exclusion, which will lead to the boundedness of the hyperchaotic system and the complexity of the motion in the bounded orbit. As shown in Figure 4, the existence of a chaotic attractor can be clearly seen from the projection of the hyperchaotic system on each plane.

3. Stability and periodicity of equilibrium for hyperchaotic system

Equilibrium of hyperchaotic system should be unstable. In the new four-dimensional hyperchaotic system, the equilibrium point is unstable when at least one of the real parts of all eigenvalues of the Jacobian matrix corresponding to the equilibrium is greater than 0. The equilibrium and corresponding eigenvalues of the new four-dimensional hyperchaotic system are shown in Table 3.

From Table 3, it can be seen that the eigenvalues of each equilibrium have positive numbers, which indicates that the equilibrium of the chaotic system is unstable.

Through the above analysis and simulation, it can be seen that the dimension of the new hyperchaotic system is greater than 4. Moreover, there are two positive Lyapunov exponents; at least two equations have at least one nonlinear term. The new hyperchaotic system has a singular attractor, dissipative structure and the instability of equilibrium. All these show that the new system is a hyperchaotic system.

#### 3.1.3. Pseudo-Random Sequence Generation

**Figure 5:**
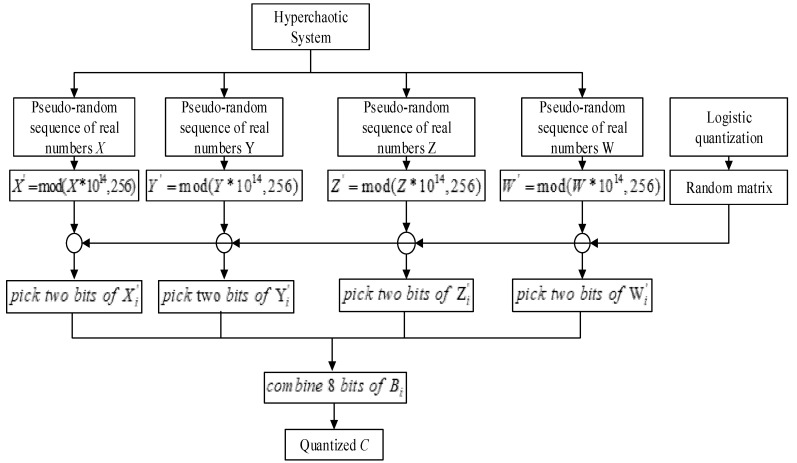
Pseudo-random sequence generation algorithm based on hyperchaotic system.

The pseudo-random sequence produced by the new hyperchaotic system needs to be quantized to satisfy the encryption operation. In this paper, a new quantization method is designed to increase the complexity of the generated sequence. The design process is shown in Figure 5.

The pseudo-random sequence generation algorithm based on hyperchaotic system is described in the following steps:

Step 1: According to the iteration of each variable of the four-dimensional hyperchaotic system, four sets of iteration values are generated: they are *X*, *Y*, *Z*, *W*;

Step 2: Perform modulo 256 operations for each real number sequence of *X*, *Y*, *Z*, *W,* as follows:

(17) $$\{\begin{array}{c} X_{i}^{’}=floor(\mathrm{mod}(X_{i}\times 10^{14},256))\\ Y_{i}^{’}=floor(\mathrm{mod}(Y_{i}\times 10^{14},256))\\ Z_{i}^{’}=floor(\mathrm{mod}(Z_{i}\times 10^{14},256))\\ W_{i}^{’}=floor(\mathrm{mod}(W_{i}\times 10^{14},256)) \end{array}.$$

Step 3: Enter the initial value of the logistic chaotic map, generate the real sequence *L*, quantize *L* from 1 to 8;

Step 4: Generate the two-dimensional random matrix. Each row of the two-dimensional random matrix is 8 non-repeating integers from 1 to 8. Five such rows are randomly generated, and the order of elements in each row is different;

Step 5: Select the *i*th row of random matrix according to the value of logistic quantization sequence L. According to the *i*th row selected of random matrix, each variable in *X*, *Y*, *Z*, *W* selects 2 bits to form a new 8-bit binary sequence.

Step 6: The 8-bit binary sequences are converted to decimal, which form an integer from 0 to 255. Thus, the quantization of the sequence is completed, and the pseudo-random sequence is generated.

### 3.2. Encryption Algorithm on Predicted Values and Final Predicted Errors

The initial GAP predicted values of pixels can influence the subsequent context processing and predicted errors can also influence entropy coding. Because predicted values and predicted errors are generated only once each time, scrambling will waste storage space, so only diffusion is performed here.

In order to compare whether the diffusion operation has a significant impact on the compression ratio, this paper designs two different encryption methods. One is the feedback diffusion method based on CBC mode, and the other is the XOR operation of plaintext. The pseudo-random sequence is produced by the new hyperchaotic system to implement encryption. In practical application, two encryption methods can be selected flexibly according to the application requirements.

1. Encryption based on CBC mode

The previous cipher text is used as the input to affect the encryption of the subsequent plaintext, and then XOR with the pseudo-random sequence produced by the four-dimensional hyperchaotic system. Diffusion operation is shown as follows.

(18) $$\{\begin{array}{c} \{\begin{array}{c} C(m)=bitxor(t,B(m))\\ R(m)=bitxor(C(m),T(m)) \end{array}\;\;\;\;\;\;\;\;\;\;\;\;\;\;\;\;\;\;\;\;\;\;\;\;\;\;\;\;m=1\\ \{\begin{array}{c} C(m)=bitxor(R(m-1),B(m))\\ R(m)=bitxor(C(m),T(m)) \end{array}\;\;\;\;\;\;\;\;\;\;\;\;\;\;\;\;m>1 \end{array}.$$

where *B* is the pseudo-random sequence produced by the four-dimensional hyperchaotic system, *R* is the cipher-text, *T* is the plaintext, *C* represents the intermediate variable of encryption. The symbol *t* is an initial random number between 0 to 255 which is used to encrypt the first plaintext.

2. Encryption based on XOR mode

In this encryption mode, the plaintext and the pseudo-random sequence are performed XOR operation directly. One bit is taken from each value, and so the eight binary bits in eight values from pseudo-random sequence form a new integer between 0 to 255. In order to increase the security, the new integer is used to perform the XOR operation. The operation is shown in Equation (19).

(19) $$\{\begin{array}{c} S_{i1}=binary((B_{i}\&1),(B_{i+1}\&2),(B_{i+2}\&4),(B_{i+3}\&8)\\ S_{i2}=binary((B_{i+4}\&16),(B_{i+5}\&32),(B_{i+6}\&64),(B_{i+7}\&128)\\ K_{i}=\mathrm{combine}(S_{i1},S_{i2})\\ C_{i}=P_{i}⊕K_{i} \end{array}.$$

where *P* stands for the plaintext, *B* represents the pseudo-random sequence based on the hyperchaotic system and *K* is a new pseudo-random sequence based on pseudo-random sequence *B*.

### 3.3. Design of Image Pixels and Entropy Coding Encryption

The two lines of image pixels are the basis of prediction. After the prediction and coding, the first two lines of pixels need to be read and encapsulated. We can perform strong encryption on the two lines of pixels.

Diffusion and scrambling are performed on two lines of pixels and the entropy coding file. Scrambling is performed based on the two-dimensional baker map.

1. Scrambling with the two-dimensional baker map

**Figure 6:**
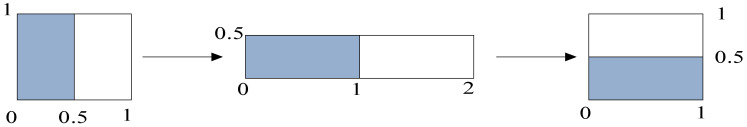
Principle diagram of two-dimensional baker map.

The transformation of the two-dimensional baker map is similar to the “kneading“ process, and the principle diagram is shown in Figure 6.

From Figure 6, permutation based on the two-dimensional baker map is designed and shown in Equation (20).

(20) $$\{\begin{array}{l} I[2i+1,2j-1]=I[i,j]\;\;\;\;\;\;\;\;\;\;\;\;\;\;\;\;\;\;\;\;\;\;\;\;\;\;\;\;\;\;\;\;\;\;\;\;\;\;\;\;\;\;1\leq i\leq m/2,1\leq j\leq n/2\\ I[2i-1,2n-2j+2]=I[i,j]\;\;\;\;\;\;\;\;\;\;\;\;\;\;\;\;\;\;\;\;\;\;\;\;\;\;\;\;\;\;\;1\leq i\leq m/2,n/2\leq j\leq n\\ I[2i-m,2j]=I[i,j]\;\;\;\;\;\;\;\;\;\;\;\;\;\;\;\;\;\;\;\;\;\;\;\;\;\;\;\;\;\;\;\;\;\;\;\;\;\;\;\;\;\;\;\;\;\;\;\;\;\;m/2<i\leq m,1\leq j\leq n/2\\ I[2i-m-1,2n-2j+1]=I[i,j]\;\;\;\;\;\;\;\;\;\;\;\;\;\;\;\;\;\;\;m/2<i\leq m,n/2<j\leq n \end{array}.$$

Based on the pixel scrambling method shown in Equation (20), set the times of scrambling, and perform scrambling.

2. Pixel diffusion with table lookup method

**Figure 7:**
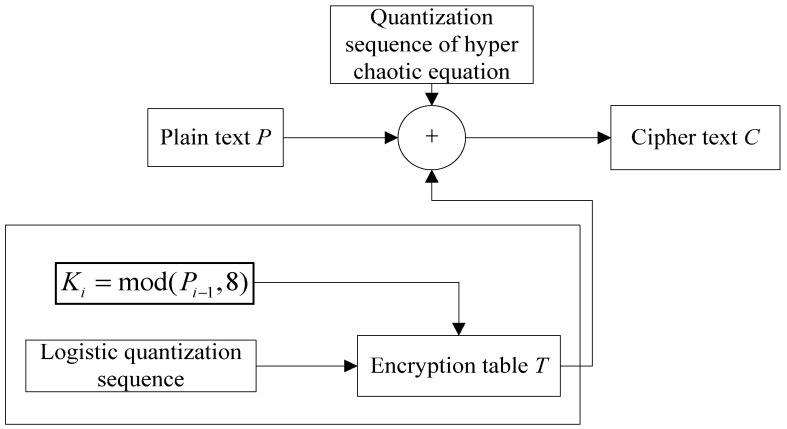
Pixel diffusion principle diagram.

In order to prevent the attack caused by the same type of operation on different pixels, a nonlinear method based on encryption operation table is designed. In the method, the encryption is related to the plaintext, which can enhance the security of diffusion operation. The principle of pixel diffusion is shown in Figure 7.

From Figure 7, cipher-text after scrambling is performed on the XOR operation with two results. The two results include the pseudo-random sequence based on the hyperchaotic system and the output result of the encryption operation table. The security of the encryption operation table has a great impact on the security of the whole diffusion system. In order to enhance the security, the table lookup and operation in the table are related to the chaotic map and plaintext. The specific encryption operation table is shown in Table 4.

From Table 4, the operation included in the non-linear operation table includes six different operations. They are bitwise NOT, recycle shift, XOR operation and the combination of different operation modes. As shown in Figure 7, the encryption really achieved the relevance to plaintext. The encryption operation is decided by the plaintext, which increases the security of the algorithm. In terms of specific operation, the pseudo-random sequence between 0 and 5 is generated by logistic map, which is used to select the entry of encryption operation table for each diffusion operation. The shift operation in the encryption operation table is also related to plaintext, and the number of bits of shift operation is determined by the result of modulo 8 operations for plaintext. Diffusion encryption based on the encryption table is shown in Equation (21).

(21) $$C_{i}=P_{i}⊕B_{i}⊕T_{i}.$$

where *C* represents cipher-text, *P* for plaintext, *B* for the pseudo-random sequence based on hyperchaotic system and *T* for output value from encryption operation table.

The image compression and encryption scheme include encryption of the four encryption locations mentioned above. Decryption operation is the reverse process of encryption operation, which will not be described in detail here. The only thing to note is that in the decryption process, the structure of the encryption operation table will be changed. For example, rotating left operation should be changed to rotating right operation, and vice versa.

## 4. Performance Analysis of Lossless Image Compression and Encryption Scheme

**Figure 8:**
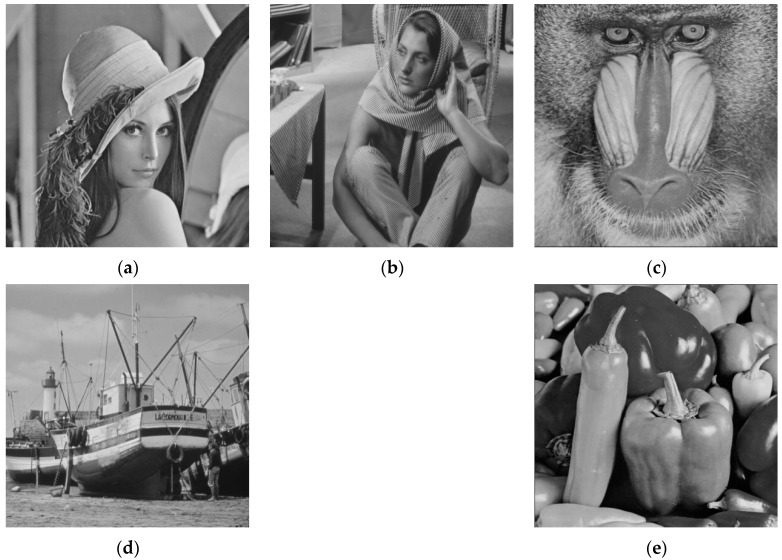
Grayscale images for testing. (**a**) Lena; (**b**) Barbara; (**c**) baboon; (**d**) boat; (**e**) peppers.

The experimental environment is Windows 7 32-bit systems, running memory is 2G, and main program running software is Visual Studio 2010 together with Opencv1.0. The test images are 512 × 512 standard test grayscale image Lena, Barbara and obtained from USC-SIPI image database, as shown in Figure 8.

Initial parameters of the four-dimensional hyperchaotic system are X0 = 0.58471298567391, Y0 = 0.36471847639187, Z0 = 0.76812659837126, W0 = 0.14587965412594. The initial parameters of the logistic map for the random matrix selection are x = 0.12145621364516. The random matrix used for constructing pseudo-random sequence is:

(22) $$\left[\begin{array}{cccccccc} 3 & 7 & 1 & 8 & 4 & 5 & 6 & 2\\ 8 & 7 & 5 & 1 & 6 & 3 & 2 & 4\\ 4 & 7 & 8 & 3 & 2 & 6 & 5 & 1\\ 8 & 2 & 4 & 5 & 3 & 6 & 1 & 7\\ 8 & 5 & 7 & 4 & 3 & 6 & 1 & 2 \end{array}\right].$$

In two lines of original pixel and entropy coding encryption, the initial value of logistic map for selecting the entry of encryption operation table is x = 0.361846592847235.

To prove the lossless property of the proposed image encryption and compression scheme, we used MSE in Equation (23) to evaluate the differences between an original image $\hat{f}(i,j)$ and the corresponding reconstructed image f(i,j).

(23) $$MSE=\frac{1}{M\times N}\overset{M}{\underset{i=1}{\sum }}\overset{N}{\underset{j=1}{\sum }}{\left(\hat{f}(i,j)-f(i,j)\right)}^{2}.$$

where *M* and *N* represent the height of image and the width of image, respectively.

Obviously, the value of MSE equaling to 0 means lossless property. Table 5 lists the value of MSE for the six images.

From Table 5, the values of the six images are all 0. This means the proposed compression and encryption scheme is lossless.

### 4.1. Compression Performance of Lossless Image Compression and Encryption Scheme

1. Compression ratio analysis

Because encryption on predicted values and predicted errors can influence compression, we compare the compression ratio of two encryption schemes proposed in Section 3.2. The comparison result is shown in Table 6. Here, the unit of compression ratio is bpp (bit per pixel) which is the required number of bits for each pixel.

As can be seen from the Table 6, encryption with XOR mode has less influence on the compression ratio than encryption with CBC mode. Although encryption with CBC mode is more secure than encryption with XOR mode, it has obvious impacts on compression ratio and does not have the advantage of the CALIC compression algorithm in the compression ratio. 

Because there are still pixel encryption and entropy coding encryption, choosing the encryption with XOR mode can make the compression and encryption algorithm more efficient.

2. Compression efficiency analysis

The time of the secure CALIC scheme includes the compression time and the encryption time of each encryption part. To improve the measurement accuracy, the compression time is measured before adding the encryption algorithm, and then the compression and encryption time is measured. Finally, encryption time and the percentage of encryption time in the total time are calculated. The running time of different images is shown in Table 7.

CALIC lossless image compression algorithm based on prediction mode only scans the image once and for one code residual, therefore, the efficiency is very high, and it is easy to implement in hardware. After the addition of the encryption algorithm, the encryption algorithm time does not greatly affect the compression time and accounts for about 20% of the total time with higher efficiency.

### 4.2. Security Analysis of Lossless Image Compression and Encryption Scheme

1. Key Space

For a good encryption algorithm, key space should be large enough to resist the brute force attack. In our algorithm, there are two keys: the pseudo-random sequence generator key and image pixels and the entropy coding encryption key.

For the pseudo-random sequence generator key, there are four real numbers for initial parameters of the four-dimensional hyperchaotic system and one real number for initial parameter of logistic map. To avoid the negative effects caused by discretization, the real numbers should be stored and transmitted using a real data type with high precision. If the implementation language complies with IEEE Standard 754-2008, then it is recommended to use the double data type. The double data type stores real numbers in 8 bytes with an accuracy of 15 decimals. Thus, the length of key will be of 320 bits, which means the size of key space will be equal to 2^320^. In addition, for a random matrix used in pseudo-random sequence generator, there are 8! = 40,320 non repetitive permutations. Five permutations are randomly selected, and there are $A_{40,320}^{5}$ selections in total.

For image pixels and the entropy coding encryption key, there is a real number for the initial parameter of logistic map used in pixel diffusion with table lookup method. Thus, the length of key will be of 64 bits, which means the size of key space will be equal to 2^64^.

Therefore, the total key space is 2^384^, together with $A_{40,320}^{5}$ random matrix space, which is strong enough to resist the brute force attack.

2. Fault tolerance of algorithm

Fault tolerance of algorithm indicates whether the compressed file of the image can be recognized by the image reader in the case of error decryption. The image compression and encryption algorithm proposed in this paper adds an encryption mechanism to the original CALIC compression algorithm. A small error occurring in the decryption process will lead to the destruction of file format so that the image reader cannot correctly recognize the image. Therefore, it can further enhance the security. We judge whether the compressed file of the image can be recognized by the image reader by introducing small changes to keys in the decryption process of different encryption locations. The results are shown in Table 8.

In Table 8, the symbol √ indicates an image file that can be recognized, symbol × indicates an unrecognized image file. As can be seen from Table 8, in the four encryption locations, only the image pixels with a wrong key will not interfere with the final integrity, which can achieve effective recognition. Predicted values, predicted errors and entropy coding encryption with the wrong key all will cause image damage which cannot be recognized by the image reader. This shows that the proposed algorithm has low fault tolerance but high security. The two lines of pixels are written to the coding file after coding and they are not involved in the coding process, so it has no effect on the file format.

3. Key sensitivity analysis

A good encryption scheme should be sensitive to the key. A tiny modification of 1 bit to the keys is used to encrypt the image. Each bit in the resultant cipher-text is compared with the corresponding bit in the cipher-text with the original key, and the number of different bits is counted. The mean values of bit change rate of the cipher-text for the different keys are shown in Table 9.

From Table 9, bit change rate of the cipher-text is about 50%. It shows that the compression algorithm can also have a strong key sensitivity after the embedding of the encryption algorithm.

4. Statistical analysis

**Figure 9:**
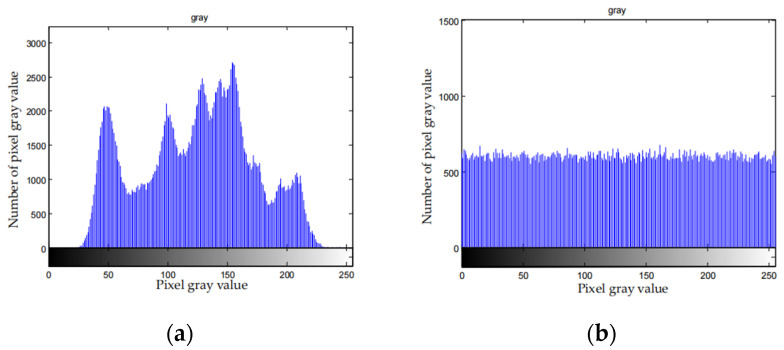

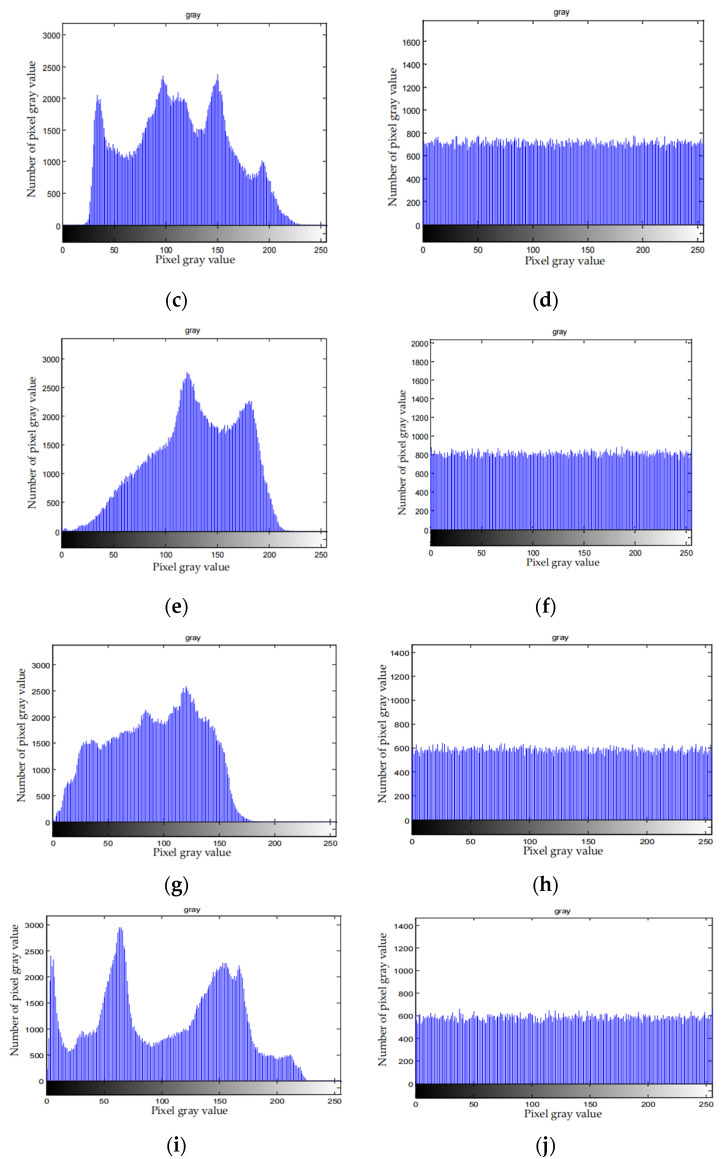
Histogram of plaintext and cipher-image of different images. (**a**) Lena plain image histogram; (**b**) Lena cipher-image histogram; (**c**) Barbara plain image histogram; (**d**) Barbara cipher-image histogram; (**e**) Baboon plain image histogram; (**f**) Baboon cipher-image histogram; (**g**) Boat plain image histogram; (**h**) Boat cipher-image histogram; (**i**) Peppers plain image histogram; (**j**) Peppers cipher-image histogram.

The pixel distribution of the image containing the valid information is not uniform. After encryption, the pixel distribution is uniform and can resist statistical analysis. Figure 9 depicts the histograms of the original images and the corresponding cipher-texts.

From Figure 9, distribution histogram is uniform after the compression and encryption. Therefore, attackers cannot attack the file with the statistical characteristics of the histogram of the cipher-text, which means the algorithm can effectively resist statistical attack.

5. Correlation Analysis

Correlation between adjacent pixels is one of the important characteristics of images that are different from other common files. Plain image has a strong correlation. A good encryption algorithm can eliminate the correlation between the adjacent elements of the encrypted file so that the value of other elements cannot be inferred through one element. Correlation calculation is shown in Equation (24).

(24) $$C_{r}=\frac{N\sum_{j=1}^{N}\left(x_{j}\times y_{j}\right)-\sum_{j=1}^{N}x_{j}\times \sum_{j=1}^{N}y_{j}}{\sqrt{\left(N\sum_{j=1}^{N}x_{j}^{2}-{\left(\sum_{j=1}^{N}x_{j}\right)}^{2}\right)\times \left(N\sum_{j=1}^{N}y_{j}^{2}-{\left(\sum_{j=1}^{N}y_{j}\right)}^{2}\right)}}.$$

where $C_{r}$ represents pixel correlation degree, *x_j_* and *y_j_* represent the gray values of two adjacent pixels and *N* represents the number of pixels in the sample. The maximum absolute value of the correlation coefficient is 1, and the minimum absolute value is 0. The lower the correlation coefficient is, the lower the correlation of the image pixels. A good encryption algorithm should remove correlation of the image pixels to improve the resistance against an attack.

**Figure 10:**
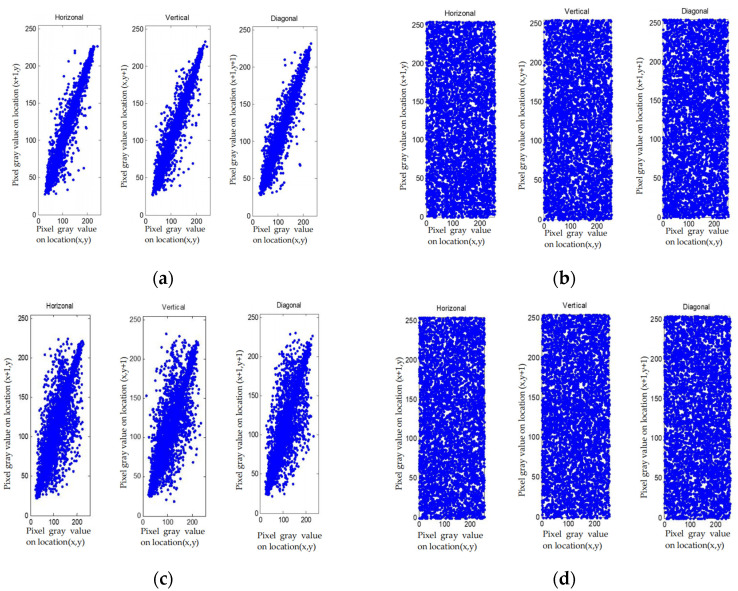

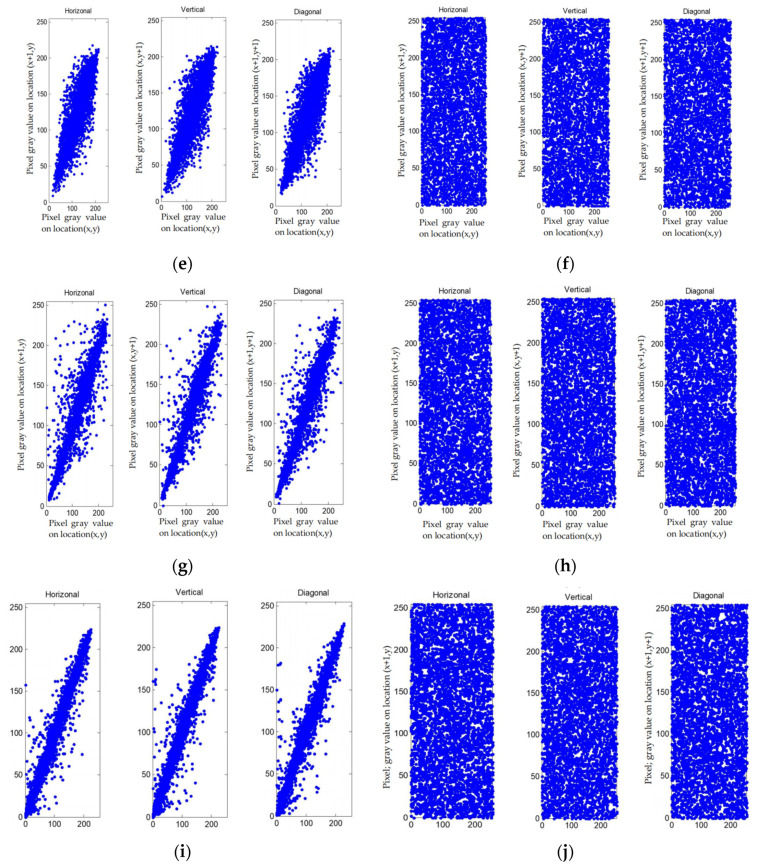
The distribution of plaintext and cipher-text of different images. (**a**) Lena plain image distribution; (**b**) Lena cipher-image distribution; (**c**) Barbara plain image distribution; (**d**) Barbara cipher-image distribution; (**e**) Baboon plain image distribution; (**f**) Barbara cipher-image distribution; (**g**) Boat plain image distribution; (**h**) Boat cipher-image distribution; (**i**) Peppers plain image distribution; (**j**) Peppers cipher-image distribution.

The pixel distribution of the plain images and cipher-images in horizontal, vertical and diagonal formats are shown in Figure 10, and the correlation test results in different directions of the test images are shown in Table 10.

According to correlation analysis from Figure 10, the distribution of the elements after being encrypted and compressed is uniform. From Table 10, correlation of plain image is large, and correlation of cipher-image is close to 0. All these indicate that the correlation between pixels is completely destroyed in the cipher-text, and we cannot obtain the pixels information via the adjacent pixels.

6. Information entropy analysis

The value of image information entropy reflects the degree of confusion. The information entropy of the encrypted file reflects the intensity and quality of the file encryption, while the information entropy of predicted errors reflects the compression ratio. The small, predicted errors indicate that the predicted errors are small and evenly distributed, which directly reflects the compression ratio and shows the advantages and disadvantages of the compression algorithm. The information entropy is shown as:

(25) $$H(x)=-\sum p(x_{i})\log_{2}p(x_{i}).$$

In Equation (25), *p*(*x_i_*) denotes the probability of symbol *x_i_*. Taking 8 bytes as a unit, if the probability of every symbol in accordance with a uniform distribution is 1/8, the entropy should be 8. A good encryption scheme should make the entropy approach 8. The entropies of the encryption file and predicted errors are shown in Table 11.

From Table 11, we can see that the information entropy of the encrypted file is close to the ideal value 8, which indicates that the encryption effect is good. Compared with this, the entropy of predicted errors is small, which indicates good compression performance.

7. Analysis of image processing attack

In the field of image processing, some image processing methods can be used to restore or improve the quality of degraded images. If image encryption is regarded as an image degradation, the plaintext image information may be obtained by using these image processing methods. Therefore, the image processing method is used to attack the cipher-image to obtain the plaintext information. Since the filter is a common method, we use four common filters to restore the Lena cipher-image, and the results are shown in Table 12.

It can be seen from the results in Table 12 that the image quality restored by these image processing methods is very low, and some are even lower than the original cipherimage, which is not helpful for image information restoration.

8. Security analysis of pseudo-random sequence

The security analysis of the pseudo-random sequence described in Section 3.1 is described below.

- Entropy test

Information entropy, approximate entropy and *K* entropy can be used to test the randomness of a pseudo-random sequence. The larger the value of information entropy, approximate entropy and *K* entropy, the better the randomness.

Information entropy indicates the confusion degree of information. The definition of information entropy is shown in Equation (25). Approximate entropy is used to calculate the probability of the new pattern in the pseudo-random sequence. The greater the probability, the larger the corresponding approximate entropy, the better the pseudo-random sequence is. Approximate entropy is shown in Equation (26).

(26) $$ApEn=2n\log_{2}\left[-(\overset{2^{m}-1}{\underset{i=0}{\sum }}\pi_{i}\log_{2}\pi_{i}-\overset{2^{m+1}-1}{\underset{i=0}{\sum }}\pi_{i}\log_{2}\pi_{i})\right].$$

where *π_i_* = *C_j_^3^*, *j* = *log_2_^i^*. *C_i_^m^* is frequency of *N* overlapping blocks.

The pseudo-random sequence to be tested is divided into innumerable small boxes, and each box contains ε value. τ represents a small time interval. $P(i_{0},i_{1},...i_{d})$ is joint probability of the value of sequence in the box of *i_0_* when τ=0 and the value of sequence in the box of *i_d_* when τ=0. The *K* entropy is defined as:

(27) $$K=-\underset{\tau \to \infty }{\lim}\underset{\varepsilon \to \infty }{\lim}\underset{d\to \infty }{\lim}\frac{1}{d\tau }\underset{i_{0},i_{1},...,i_{d}}{\sum }P(i_{0},i_{1},...,i_{d})\ln P(i_{0},i_{1},...,i_{d}).$$

The approximate entropy, information entropy and *K* entropy of pseudo-random sequence generated by the logistic map are compared with the pseudo-random sequence proposed, as shown in Table 13.

From Table 13, the pseudo-random sequence used in this paper is better than the pseudo-random sequence generated by the logistic map except that the information entropy of the pseudo-random key stream length of 800 is slightly less than that of the logistic map. It shows good randomness.

- Autocorrelation test

Autocorrelation test is an important indicator of the randomness of the pseudo-random sequence. Specific definition of sequence correlation is shown in Equation (28).

(28) $$\psi (l_{1},l_{2})=\frac{A-D}{N}.$$

where *l*_1_ and *l*_2_ represent the two pseudo-random sequences, respectively. *A* is the number of the same bit in *l*_1_, *D* is the number of the same bit in *l*_2_ and *N* represents the total length of the key stream sequences.

If *l*_1_ and *l*_2_ are the same sequence, ψ is called autocorrelation. The best state of ψ is close to a horizontal line. If the test result is a horizontal line close to 0, it shows that the test sequence has a good randomness.

**Figure 11:**
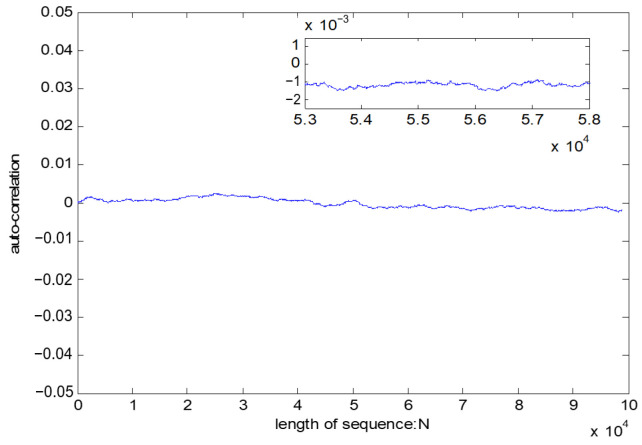
Autocorrelation test on pseudo-random sequence.

The test result of the pseudo-random sequence is shown in Table 14. The test result from Figure 11 indicates that the test result is a horizontal line close to 0, which shows a good autocorrelation of pseudo-random sequence.

- Balance test

**Figure 12:**
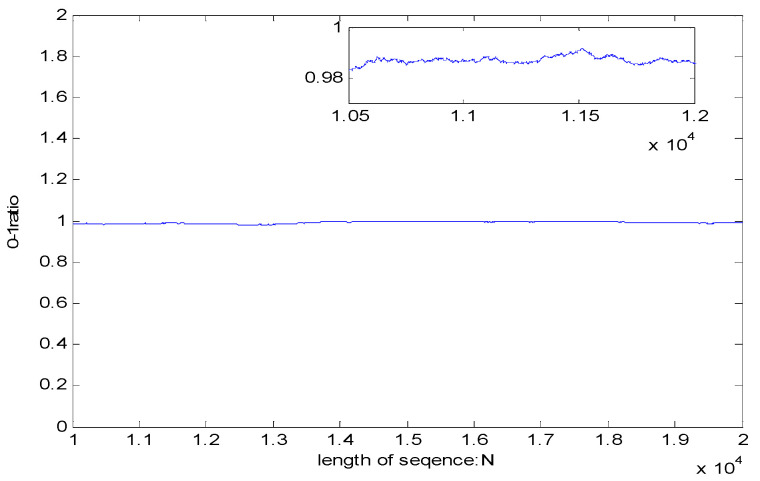
Balance test.

The balance test is used to count the ratio of 0 to 1 in the pseudo-random sequence. Ideally, the ratio of 0 to 1 is 1. The balance test is expressed as follows:

(29) $$\sigma =\frac{Sum(0)}{Sum(1)}.$$

where *Sum*(0) represents the total number of 0 in sequence, and *Sum*(1) represents the total number of 1 in sequence. The results of balance test are shown in Figure 12.

The results show that the 0, 1 distribution curve of pseudo-random sequence is close to 1, and the distribution is relatively uniform.

- Sequence distribution test

**Figure 13:**
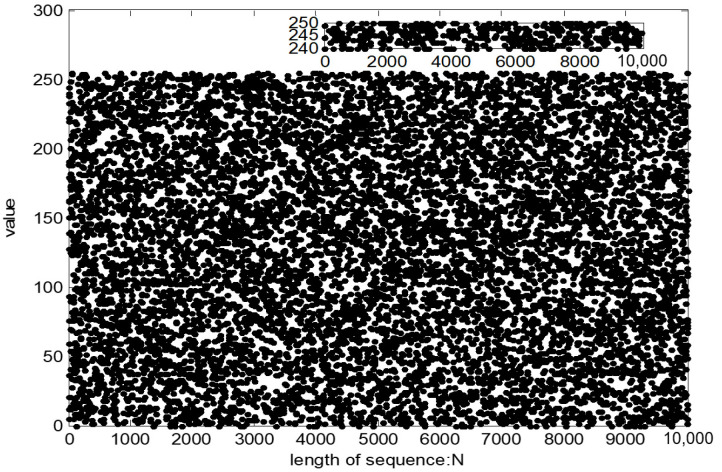
The distribution of the pseudo-random sequence.

The sequence distribution reflects the distribution of the pseudo-random sequence value. The more uniform the sequence distribution is, the better the randomness of the sequence is. As shown in Figure 13, the distribution of sequences is relatively uniform, and there is no large-scale sequence aggregation phenomenon, indicating that the randomness of the pseudo-random sequence is good.

- NIST SP800-22 tests

NIST SP800-22 tests are a testing standard to detect the deviation of a sequence from a true random sequence. It is issued by the American National Standardization Technical Committee and provides 15 methods for testing statistical characteristics. For each test, if the P-value is greater than a predefined threshold α, then it means the sequence passes the test. Commonly, α is set to 0.01. In this paper, 100 groups of sequence of 10^6^ bits are tested. Test results are shown in Table 14.

Table 14 shows that the pseudo-random sequence has a good randomness and a high security.

9. Time complexity analysis

The lossless image compression and encryption scheme are performed on gray image with *N* pixels. The time complexity of the algorithm is shown in Table 15.

According to Table 15, we can see that the time complexity of the CALIC algorithm is only related to the number of pixels that need to be scanned, and it is linear. Four encryption algorithms are stream ciphered based on different data, so the time complexity is linear. In general, the algorithm has low time complexity and high efficiency.

### 4.3. Performance Comparison with Other Schemes

In this section, we provide performance comparisons with Huffman coding and some recent works [7,8,20,26,39,44,47]. Table 16 shows the comparison results for the information entropy and compression performance.

Refs. [7,8] are image encryption schemes. Compared with the results of the information entropy in Refs. [7,8], our scheme shows superior performance. 

In Ref. [20], compression and encryption are performed separately. Ref. [26] is a joint compression and encryption scheme based on arithmetic coding. Compared with Refs. [20,26], our proposed scheme shows superior performance regarding compression performance. 

Refs. [39,44] propose a lossless image compression and encryption method. Compared with Refs. [39,44], our proposed joint lossless image encryption and compression scheme shows superior performance regarding information entropy and compression performance. 

From Table 16, although the proposed algorithm has influence on the CALIC compression ratio, it still better than compression algorithms of Huffman coding and those mentioned in Ref. [47] for the compression ratio.

## 5. Conclusions

In this paper, we first analyzed the CALIC scheme and then found four encryption locations according to the CALIC compression algorithm. The four locations in CALIC are encrypted, respectively, as: the predicted values, predicted errors, pixels and entropy coding file. In order to obtain better security, a new four-dimensional hyperchaotic system was constructed. Based on the new hyperchaotic system, a pseudo-random sequence generation algorithm used in four locations encryption was designed, and the security of the sequence was tested. According to the different characteristics of the four encryption locations, CBC mode encryption, XOR mode encryption, scrambling with the two-dimensional baker map and plaintext-related encryption based on table lookup were all used to enhance the security. For encryption based on table lookup, the operation is selected from encryption operation table based on the sequence generated by the logistic map. Each operation is related to plaintext, which can improve the security of the encryption algorithm. The algorithm was tested on compression and security performance. The experiment results show that the proposed algorithm has better security and compression performance. The scheme was tested for grayscale images. In the future, we aim to extend it to color images.

## Figures and Tables

**Table 1:** Feasibility of encryption locations.

Encryption Location	The Feasibility of Encryption	Impact on Compression Ratio	Implementation of Encryption
Plaintext image	√	Significant impact	×
The predicted values of pixels	√	Depend on encryption strength	√
The final prediction error	√	Depend on encryption strength	√
Two lines of pixel values	√	No impact	√
Compressed file after entropy coding	√	No impact	√

**Table 2:** Lyapunov exponent comparison with other chaotic systems.

Function	Lyapunov1	Lyapounov2	Lyapounov3	Lyapounov4
Proposed system	2.404	0.302	0.00	−17.534
Lorenz system	1.497	0.00	−22.46	——
Rossler system	0.112	0.019	0	−25.188
Ref. [12]	0.456	0.219	0	−15.060
Ref. [13]	2.253019	1.406374	0.054342	−38.339706
Ref. [15]	0.81	0.31	0	−24.11
Ref. [46]	0.5697	0.0453	0	−12.6078

**Table 3:** Equilibrium and corresponding eigenvalues of hyperchaotic system.

Equilibrium	Eigenvalues 1	Eigenvalues 2	Eigenvalues 3	Eigenvalues 4
(0, 0, 0, 0)	−34.68	22.94	0.38	−3.5
(−37.87, −9.15, −7.48, 46.16)	−62.38	0.29	23.56 + 38.04i	23.56–38.04i
(36.50,8.82,7.17,46.11)	−58.89	0.29	21.87 + 38.88i	21.87–38.88i

**Table 4:** Encryption operation table.

Operation Number	Encryption Operation
0	$P_{i}=\sim P_{i}$ (invert by bit)
1	$P_{i}=rollerleft(P_{i},K_{i})$ (rotate left $K_{i}$ bits of *P*)
2	$P_{i}=rollerright(P_{i},K_{i})$ (rotate right $K_{i}$ bits of *P*)
3	$P_{i}=P_{i}⊕K_{i}$ (XOR $K_{i}$)
4	$P_{i}=rollerleft(P_{i},K_{i})⊕123$ (rotate left $K_{i}$ bits of *P* and do XOR operation with constant)
5	$P_{i}=rollerleft(P_{i},K_{i})⊕35$ (rotate right $K_{i}$ bits of *P* and do XOR operation with constant)

**Table 5:** The value of MSE for the six images.

Image	Lena	Baboon	Barbara	Boat	Peppers
MSE	0	0	0	0	0

**Table 6:** Comparison of compression ratio for different encryption algorithms.

Encryption Mode	Lena	Baboon	Barbara	Boat	Peppers
No encryption	4.2812	6.0000	4.6563	4.3438	4.5625
Encryption with XOR mode	4.7186	6.3438	5.5625	4.5625	4.8438
Encryption with CBC mode	6.5938	7.0938	6.9688	6.4375	6.4375

**Table 7:** Compression time and encryption time of different images.

Time(s)	Lena	Barbara	Baboon	Boat	Peppers
Compression time	0.218	0.234	0.265	0.218	0.218
Encryption time	0.078	0.078	0.062	0.078	0.063
Total run time	0.296	0.312	0.327	0.296	0.281
Encryption time/Total run time	26.35%	25%	18.96%	26.35%	22.42%

**Table 8:** Fault tolerance of algorithm.

Encryption Location	Predicted Values	Predicted Errors	Image Pixels	Entropy Coding
Lena	×	×	√	×
Barbara	×	×	√	×
Baboon	×	×	√	×
Boat	×	×	√	×
Peppers	×	×	√	×

**Table 9:** Key sensitivity test.

Image Name	Lena	Barbara	Baboon	Boat	Peppers
Change rate of cipher text	0.49944	0.49963	0.49975	0.49993	0.49967

**Table 10:** Correlation coefficient of plain image and cipher-image.

Image		Horizontal Direction	Vertical Direction	Diagonal Direction
Lena	Plain image	0.9722821	0.9852186	0.9608765
	Cipher-image	0.0116133	0.0125545	0.0218114
Barbara	Plain image	0.8612652	0.9595282	0.8468656
	Cipher-image	0.0122084	0.0079887	0.0200846
Baboon	Plain image	0.8689049	0.7629869	0.7403367
	Cipher-image	0.0071669	0.0115003	0.0181280
Boat	Plain image	0.9635128	0.9789292	0.9478472
	Cipher-image	0.0063547	0.0098399	0.0221604
Peppers	Plain image	0.9793075	0.9827495	0.9685861
	Cipher-image	0.0110749	0.0073561	0.0194759

**Table 11:** Information entropy.

		Lena	Barbara	Baboon	Boat	Peppers
Information entropy	Original image	7.4483	7.4664	7.3579	7.1237	7.5714
	Encryption file	7.9989	7.9901	7.9987	7.9989	7.9988
	Final prediction error	4.2812	6.0000	4.6563	4.3438	4.5625

**Table 12:** Image processing attack.

Test Item	Lena Cipher-image	Mean Filter	Median Filter	Fuzzy Contrast Enhancement Filter	Wiener Filter
PSNR	5.22	6.43	5.56	4.18	6.74
MSSIM	0.011	0.019	0.008	0.008	0.020

**Table 13:** Comparison about approximate entropy, information entropy and *K* entropy of pseudo-random sequence.

Sequence Length N	Test Item	Approximate Entropy	Information Entropy	K Entropy
800	Logistic	0.6932	2.8266	0.6950
	Proposed algorithm	0.6963	2.8261	0.6980
1000	Logistic	0.6946	2.8174	0.6958
	Proposed algorithm	0.6962	2.8364	0.6976
2000	Logistic	0.6928	2.8210	0.6935
	Proposed algorithm	0.6942	2.8297	0.6949

**Table 14:** SP800-22 tests for the pseudo-random sequence.

Statistical Test	*p*-Value	Proportion
Frequency	0.494392	0.9800
Block Frequency	0.574903	0.9900
Cumulative Sums	0.955835	0.9900
Runs	0.779188	0.9900
Longest Run	0.319084	0.9800
Rank	0.574903	0.9900
FFT	0.102625	0.9900
NonOverlappingTemplate	0.484732	0.9800
Overlapping Template	0.470723	1.0000
Universal	0.759756	0.9900
Approximate Entropy	0.437274	0.9800
Random Excursions	0.657933	0.9921
Random Excursions Variant	0.383827	0.9932
Serial	0.987896	0.9900
Linear Complexity	0.249284	0.9700

**Table 15:** Time complexity of proposed algorithm.

Operation Item	Time Complexity
CALIC compression algorithm	*O*(*n*)
GAP predicted pixel encryption	*O*(*n*)
Predicted errors encryption	*O*(*n*)
Image pixels encryption	*O*(*n*)
Entropy encoding encryption	*O*(*n*)

**Table 16:** Performance comparison.

Test Images	Scheme	Information Entropy	Compression Ratio
Lena	Our scheme	7.9989	4.7186
	Ref. [7]	7.9896	N/A
	Ref. [8]	7.9972	N/A
	Ref. [20]	N/A	4.878
	Ref. [26]	N/A	6.912
	Ref. [39](128 × 128 encrypted block)	7.9887	5.6843
	Ref. [44] (128 × 128 encrypted block)	7.5802	6.4449
	Ref. [47]	N/A	7.1
	Huffman coding	N/A	7.8

## Data Availability

Additional data have not been reported.
